# Hospice de Chinon

**Published:** 1832-09

**Authors:** 


### HOSPITAL REPORTS.

### HOSPICE DE CHINON.

### *Complete Imperforation of the Vagina, successfully treated by* MM. DESMÉE *and* GENDRON.*

MARIE TAFFENEAU, seventeen years of age, fair, fresh-coloured, of a strong constitution, and accustomed to field labour, had been ailing for about six months with uneasy sensations in the abdomen, which during that period gradually increased in bulk; when she applied for admission into the hospital, and was received on the 16th of March, 1831.

She was examined, in consultation, by Drs. LAFON, senior and junior, and M. DESMÉE, at the request of M. GENDRON, the surgeon, under whose care she was placed.

The abdominal enlargement had much the appearance of a five-months' pregnancy; for the tumor had risen out of the pelvis, and extended as far as the navel. Examination per vaginam was attempted; but, to the surprise of the surgeon, the passage was completely impervious, a thick rigid membrane so entirely closing the vagina that not even a sound could be introduced. +

It was evident from this, that the case was one of retention of the menses, and the girl confessed that she had never been unwell after the manner of women. On separating the labia majora, a tumor, the size of a two franc-piece and of a roundish form, was perceived; which, when pressed upon by the index finger, while the other hand made counter pressure on the abdomen, gave a very decided sense of fluctuation; and when the forefinger was introduced into the rectum, the thumb of the same hand being placed upon the swelling in the vagina, the fluctuation in the tumor was still more apparent.

These circumstances determined the surgeons consulted to recommend the operation for imperforate vagina; but, nevertheless, the excellent health which the girl had hitherto enjoyed, even during the time that the tumor had been forming, caused the only means

* Gazette Medicale.

+ How did she make water?

which the symptoms indicated for her relief to be postponed for several days; yet, on a second consultation, it was decided to operate without further delay; and M. Gendron opened the tumor with a bistoury, to which instrument he gave the preference. Immediately the puncture was made, a large quantity of a red, indorous, slightly viscid fluid escaped. The swelling of the abdomen at once subsided; the patient did not seem at all faint from the sudden discharge, but declared herself much relieved. Injections of warm water and the use of the hipbath were prescribed; and the discharge entirely ceased within twenty-four hours.

The patient might have quitted the hospital at once, but she was detained for the purpose of ascertaining whether the regular menstrual discharge would take place. This occurred sixteen days after the operation; but the quantity was small, and it only lasted twelve hours. She left the house three days afterwards, but since then it has been learned that her courses are regular, and the discharge abundant.

---

